# Prevalence of depression in people with tuberculosis in East Africa: a systematic review and meta-analysis

**DOI:** 10.4314/ahs.v23i1.25

**Published:** 2023-03

**Authors:** Wondale Getinet Alemu, Tadele Amare Zeleke

**Affiliations:** Department of Psychiatry College of Medicine and Health Science, University of Gondar, Gondar, Ethiopia

**Keywords:** Depression, East Africa, meta-analysis, systematic review, TB patients

## Abstract

**Background:**

Depression is one of the most common mental health problems comorbid with tuberculosis. However, a consolidated picture of the prevalence of depression among tuberculosis patients in East Africa remains unknown. This systematic review and meta-analysis provide new understandings by systematically examining evidence concerning the prevalence of depression among tuberculosis patients in East Africa.

**Methods:**

Literature was found in a database of HINARI, SCOPUS, PubMed, Science Direct, and Google Scholar. The Newcastle-Ottawa quality assessment scale was used to appraise the quality of the selected studies. Then, the DerSimonian and Laird random-effects model was applied because of the presence of heterogeneity among studies.

**Results:**

A total of 409 studies were accessed. However, only 29 qualified for a full-text review, and 9 studies with a population of 2838 were included in the qualitative description and quantitative analysis. The pooled prevalence estimate of depression amongst tuberculosis patients was 43.03 % (34.93, 51.13). The highest prevalence was observed in Kenya, with 45.71% (29.26, 62.16); a similar rate was observed in Ethiopia, with 45.11 % (34.60, 55.62). Subgroup analysis based on screening tool was used: 45.71% with BDI and 41.53% with PHQ.

## Background

Depression, often known as major depressive disorder or clinical depression, is a significant mood condition that affects many people. Depression causes people to feel unhappy and hopeless all the time, and they lose interest in activities they used to enjoy. Aside from the emotional concerns that depression causes, people may also have physical symptoms such as persistent pain or digestive problems. Symptoms of depression must be present for at least weeks in order to be diagnosed 1. Depression is one of the most common mental disorders. It is characterized by sadness, loss of interest or pleasure, feelings of guilt or low self-esteem, disturbed sleep, or appetite, decreased energy, ad poor concentration. These factors are experienced by most people at certain points in life and can also manifest as mourning, hyperactivity, and manic behaviour 2. Today, depression is the leading cause of poor health and burden worldwide, with more than 300 million people affected an increase of more than 18% between 2005 and 2019 3.

Tuberculosis is a chronic multisystem infectious disease caused by the mycobacterium tuberculosis bacteria. Tuberculosis is one of the leading causes of morbidity and mortality globally 4–6. The prevalence of mental disorders, including depression and anxiety disorders, among people with TB, is estimated to be between 40% and 70% 7–9. “Depression has been an invisible burden for people with TB. The risk of people with mental disorders being prone to developing TB, or the mental well-being of TB patients during their treatment, has often been overlooked”^10^. The link between tuberculosis and mental illness is complex, and they share common risk factors like homelessness, HIV-positive status, and substance use. These, in turn, lead to frequent co-morbidity^11^. Depression and tuberculosis alone contribute 2.5% and 2.0% to the global disease burden, respectively^12^. Depression appears to be highly prevalent in people with TB^13^–^15^. A Global Burden of Disease (GBD) study found that depression is the fourth greatest cause of global disability-adjusted life years (DALY)^16^, But, little study has been done on the link between tuberculosis and mental illnesses^17^.

However, though it is one of the most common co-morbidities in patients with tuberculosis, the causes of this co-morbidity remain unclear.

Depression is highly prevalent in adults with tuberculosis and is associated with worse health status in comparison to tuberculosis without depression 18. Depression is linked with greater morbidity, mortality 19, 20, drug resistance^21^, and community transmission 22. Thus, considering and addressing the social and unusual complexities of TB and mental illness is critical to reducing the rates of TB and its complications globally.

Some studies have been conducted on the prevalence and associated factors of depression amongst tuberculosis patients. However, the findings of these analyses indicated differences in prevalence and associated factors. Therefore, this systematic review and meta-analysis aimed to estimate the pooled prevalence of depression amongst TB patients in East Africa.

## Methods and Analysis

### Protocol registration and review reporting

This study has been registered at PROSPERO, the international prospective register of systematic reviews and meta-analysis (registration number CRD-42019121256). The PRISMA checklist was employed to prepare the protocol. Furthermore, to report the findings of the analysis, the meta-analysis of observational studies in epidemiology (MOOSE) guidelines for reporting 23 and the PRISMA reporting checklist 2009 were utilized 24. To demonstrate the procedure of the screening and selection processes, a PRISMA flow diagram was used. The findings of the meta-analysis study are presented through figures and texts.

### Eligibility criteria

All references to relevant articles which was done on tuberculosis adult patient who are on follow up and screened positive for depression were eligible and articles are included. All articles irrespective of year of publication included.

### Data sources and search strategy

To access published studies, PubMed, SCOPUS, HINARI, Science Direct, and Google Scholar were used. All references to relevant articles were followed to access other additional studies. Furthermore, the corresponding authors were contacted via email or other means of communication for articles that were difficult to access and for other necessary information. The key search terms were “depression on TB” or “Tuberculosis and depression”, “determinant factors”, “associated factors” and east African countries (“Ethiopia” or “Eritrea” or “Kenya” or “Uganda” or “Tanzania” or “Sudan” “Djibouti” or “Somalia” or “Rwanda”). All these terms were searched using the advanced search function of the databases through “MeSH terms” and “All fields” by connecting the “AND” and “OR” Boolean operators, as appropriate. These activities were conducted from 20/05/2019 and the study was completed by 17/09/2019.

### Inclusion and exclusion criteria

Studies that are cross-sectional methods were considered. All articles written in the English language were included. If articles reported on the prevalence and associated factors of depression amongst TB patients were included without time boundary. Articles with missing full information and difficult data extraction, even after contacting the authors with any means of communication were excluded.

### Study screening and selection

Initially, all articles accessed from the databases and search engines were exported to EndNote version 7, duplicates were identified and removed. Second, the remaining articles were evaluated based on the topic, language, and study area. Third, studies conducted globally, studies that were not in the English language, and studies on irrelevant topics were excluded. Finally, the abstracts and full texts of the remaining articles were reviewed.

### Risk of bias and quality assessment

For quality assessment, we used the Newcastle-Ottawa scale (NOS) to the studies included in the final analysis^25^. Based on our evaluation all nine studies were of good methodological quality. A review was done by two independent reviewers to assess the quality of the papers. Whenever there are inter-rater disagreements that happened between the reviewers, a thorough evaluation of sources of discrepancy was assessed and agreed upon. If disagreement persists, the average of the two reviewers calculates. After converting scores into percentages, only studies scored ≥50% were included in the systematic review as well as a meta-analysis of the prevalence.

### Data collection process

Once the eligible studies are identified, two independent reviewers extracted the data by using a prepared format on the Microsoft Excel spreadsheet. variables that were extracted are the author's name, sample size, response rate of the studies, year of the study done/publication year, a region where the study was conducted, religion, ethnicity, sex of the participants, and other important factors. For prevalence studies, the prevalence, logarithm of the prevalence, standard error of the logarithm of the prevalence was computed. For predictors, important variables of the odds ratio, logarithms of the odds ratio, and the standard error of the logarithms of the odds ratio were calculated. For any difficulties faced during data extraction, primary authors were contacted by any means of communication.

### Outcome variable

Depression is the proportion of TB patients who are positive with different screening tools for major depressive disorder.

### Assessment of publication bias and data analysis

After necessary data are extracted and documented in the Microsoft Excel spreadsheet, it was exported to STATA version 14 for further analysis. The presence of heterogeneity among studies was examined using the I^2^ heterogeneity and Q test 26. I^2^ heterogeneity test of >50% was declared as the presence of heterogeneity. Thus, the random effect model was used due to the presence of heterogeneity. To identify the influential article, sensitivity analysis is done, and treatment is applied as per result. Subgroup analysis was employed based on the country. Small studies publication bias was detected by using the symmetry of the funnel plot and objectively, through Egger's regression test 27. Asymmetry of the funnel plot or statistical significance of Egger's regression test (p-value<0.05) is suggestive of publication bias. Using the random effect model, the pooled prevalence, as well as the pooled odds ratio reported at 95% CI. If conducting a meta-analysis is a problem associated with substantial heterogeneity, results were presented using narrative synthesis.

## Results

**Fig 1:**
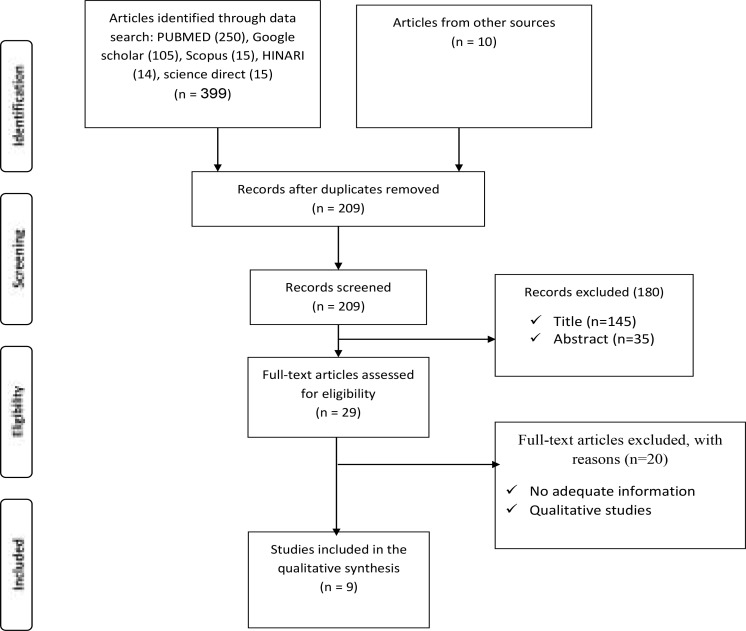
The flow chart describes the selection of studies for the systematic review and meta-analysis of prevalence and risk factors of depression among Tb patients, 2019.

Search outcomes: Literature search produced 409 articles. Among 409 articles, after duplicate removed was 209. A 180 were not fitted with the title, abstract, and inclusion criteria. Twenty papers were full of articles excluded with reasons. Lastly, 9 articles were used for synthesis included in the pooled prevalence of depression among TB patients (Figure 1).

### Features of studies

All studies were conducted in east Africa. Among the total of nine studies included in our analysis four of them were done in Ethiopia 13, 14, 23, 24, while three of the studies were conducted in Kenya 25–27, and two studies were conducted in Tanzania and Uganda respectively 28, 29. There were no time restrictions during the database search. All of them were cross-sectional surveys and no other type of study conducted on tuberculosis patients were included (Table 1).

**Table 1:** Descriptive summary of 2 studies reporting the prevalence of depression among tuberculosis patients included in the systematic review and meta-analysis, 2019

Author	Year	Study area	Sample size	The objective of the study	Prevalence (95% CI)
Dasa TT	2019	Ethiopia	403	Prevalence and associated factors of depression among tuberculosis patients in Eastern Ethiopia 23	51.9(50.02,53.77)
Ambaw F	2017	Ethiopia	657	Burden and presentation of depression among newly diagnosed individuals with TB in primary care settings in Ethiopia 13	54(52.59, 55.40)
Alemayehu M	2018	Ethiopia	415	Suicidal Ideation, Attempt, and Associated Factors among Patients with Tuberculosis in Ethiopia 24	31.1(26.69, 35.51)
Duko B	2014	Ethiopia	417	Prevalence and correlates of depression and anxiety among patients with Tuberculosis at Wolaita Sodo 14	43.4(41.2, 45.6)
Singh L	2015	Kenya	100	Psychiatric morbidity in patients of pulmonary tuberculosis-an observational study 27	44(39.56, 48.43)
Amuthonl	2008	Kenya	97	Prevalence of depression among TB patients attending TB clinic at Mbagathi District hospital Nairobi, Kenya 26	61 (51.24, 70.76)
Esther L	2015	Kenya	51	Prevalence of Depression among Active TB and TB/HIV Patients in Kisumu County 25	31.00 (18.31, 43.69)
Buberwa G	2013	Tanzania	390	prevalence of depression among tuberculosis patients attending clinics in Temeke municipal, Dar es salaam, Tanzania 28	46.9(44.8, 49)
Alinaitwe R	2018	Uganda	308	prevalence and factors associated with depressive illness in patients with tuberculosis in Mulago hospital 29	23.7(19.01,28.38)

### The pooled prevalence of depression among tuberculosis patients

**Figure 2:**
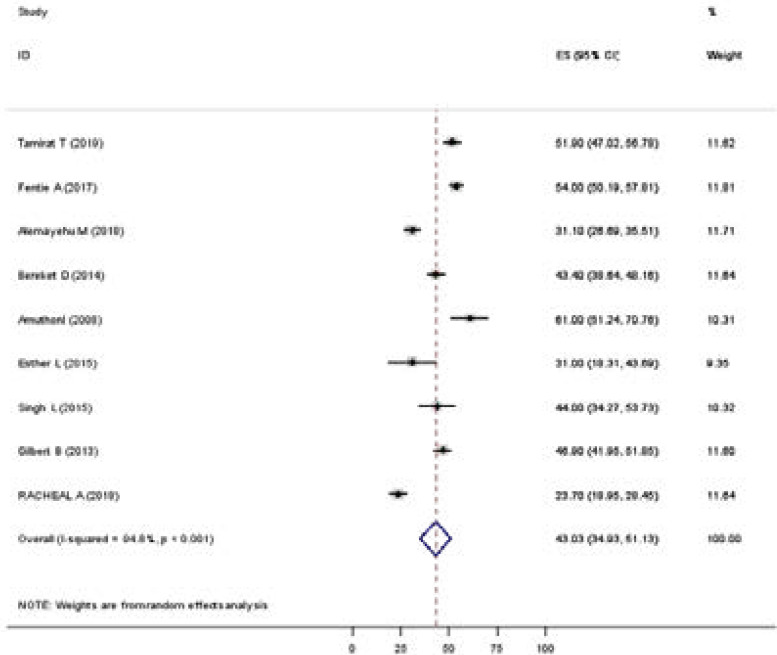
Forest plot of pooled prevalence of depression among TB patients, 2019(n=9).

The pooled prevalence of depression was 43.03 (34.93, 51.13). Heterogeneity was observed across the studies (I2 = 94.8%, p < 0.001)) (Figure-2).

**Figure 3:**
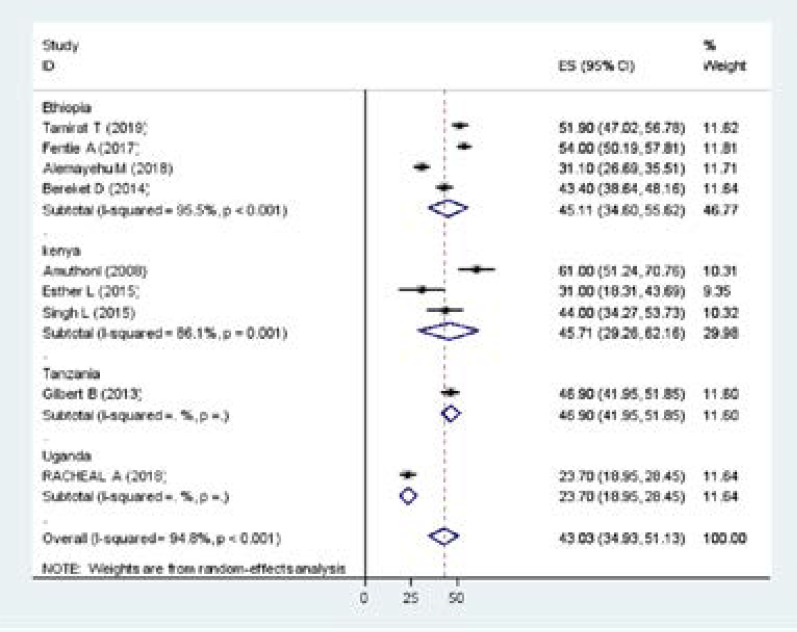
Subgroup analyses of the prevalence of depression among Tb patients, 2019(n=9)

**Figure 4:**
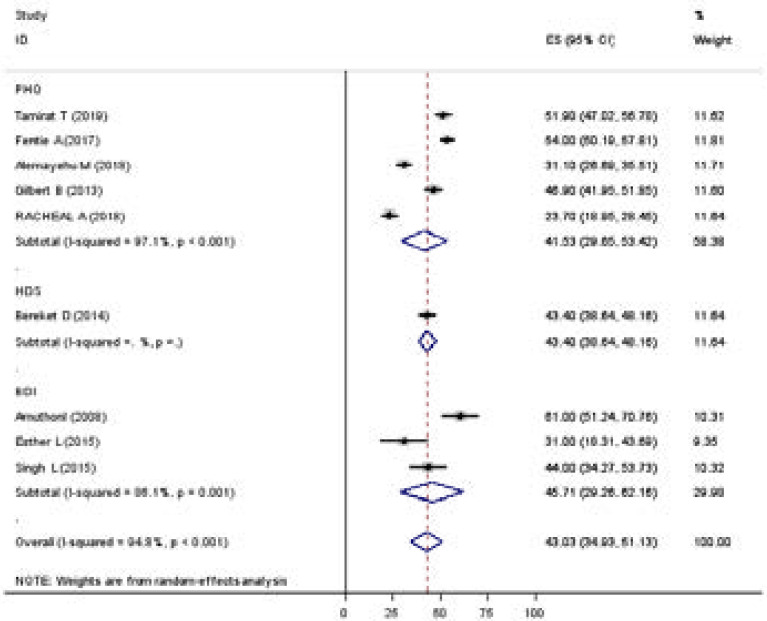
Subgroup analyses of the prevalence of depression among Tb patients, with depression screening tool 2019(n=9).

In the random-effect model, the subgroup analyses by the country where studies were done showed that the highest prevalence was observed in Kenya with 45.71% (29.26, 62.16), and similar in Ethiopia with the prevalence of 45.11% (34.60, 55.62) (Figure-3). Based on tools which depression measured PHQ 41.53 (29.65, 53.42) &BDI 45.71 (29.62, 62.16) (Figure-4).

**Figure 5:**
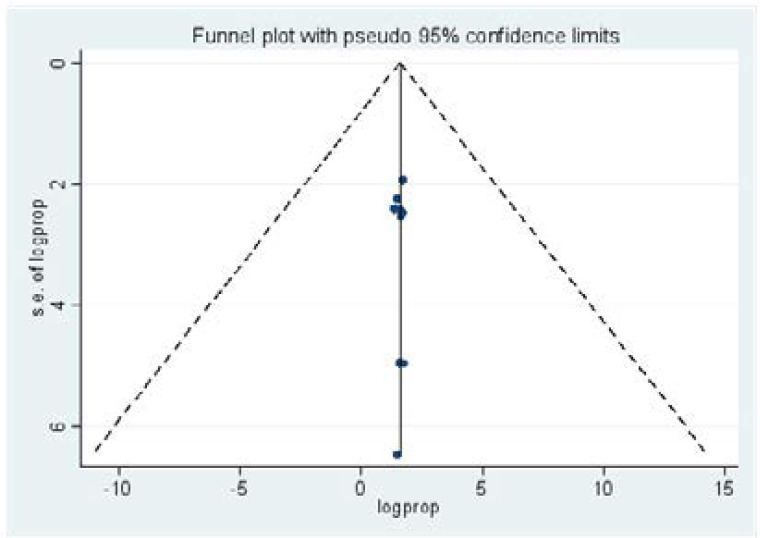
Funnel plot showing publication bias of prevalence studies among TB patients, a systematic review and meta-analysis, 2019, (n=9).

Publication bias: The funnel plot and Egger's regression tests (B= -0.0002, SE=0.055, P = 0.99) show there was no evidence of bias upon observation of funnel plots and Egger's regression test (Fig-5).

## Discussion

In our context to the best of our knowledge, this systematic review and meta-analysis are the first of their kind on the assessment of the pooled prevalence of depression on tuberculosis patients in East Africa. The key aim of the study was the identification of epidemiological information on the overall prevalence of depression in tuberculosis patients in east Africa. It also helps that the results of this review provide greater evidence than the results of the studies separately done.

Nine studies that examined the prevalence of depression and associated factors in tuberculosis were included.

The pooled prevalence of depression was found to be 43.03 (34.93, 51.13). The prevalence of depression on patients with tuberculosis is different in magnitude in the country where the studies have been done as well as the tool used to measure depression. The highest prevalence was observed in Kenya with 45.71% (29.26, 62.16), and similar in Ethiopia with a prevalence of 45.11% (34.60, 55.62). The pooled estimate prevalence of the depressive disorder in patients with tuberculosis in our systematic review and meta-analysis has a big difference when compared with the 4% prevalence, found in the general population^30^. This systematic review and meta-analysis are in line with a study by Duko B, 2020 among tuberculosis patients who had depressive disorder 45.19%^31^, but it was higher than the study done in sub-Saharan Africa pooled prevalence of depression among patients with tuberculosis was 39.42%^32^.

Of course, the prevalence of depression revealed a significant difference depending on the measurement tool used to determine the magnitude of depression in patients with tuberculosis. The pooled estimate prevalence of depression in patients with tuberculosis was higher in studies conducted using screening tools than diagnostic instruments as expected. The estimated pooled prevalence of depression in patients with tuberculosis was found to be 45.71%, 41.53% as screened by BDI, PHQ, respectively. The reason for a diagnostic tool providing more emphasis to high specificity as related to screening instruments that focus on sensitivity might be the reason for the observed difference. The other possible explanation might be diagnostic instruments uses strict criteria as compared to screening instrument as screening tool intended to detect potential indicators for illness or possible illness rather than finding the presence or absence of disease is the main purpose of diagnostic instrument. Finally, the possible explanation for the difference in the prevalence of depression among the different screening instruments may be due to differences in the sensitivity and specificity of the tools used to screen depression in patients with tuberculosis. The results support the view that validation and use of the standard instrument for screening as well as diagnosis of depression in patients with tuberculosis.

Regarding determinant factors, our meta-analysis synthesis indicated that factors such as female sex, being in intensive phase treatment of tuberculosis, family history of depression, level of education, comorbid HIV infection, poor social support, perceived stigma, physical illness, having night sweating and old age were associated with depression in people having tuberculosis in east Africa. This finding provides evidence on the prevalence of depression among tuberculosis patients in east Africa with relevant data. Measures to lessen the severity of depression should be addressed and the following priority areas must be considered: early detection, prevention, and intervention.

## Conclusions

Patients with tuberculosis appeared to have a significant rate of depression. Even though we did not conduct pooled effect size of different factors, female sex, tuberculosis in the severe phase of treatment, family history of depression, level of education, concomitant HIV infection, limited social support, perceived stigma, physical disease, nocturnal sweating, and old age should all be considered risk factors from every single study.

According to a pooled estimate, depression was shown to be common among tuberculosis patients, as a result, it is suggested that mental health screening and integration measures be implemented.

## Limitations of the study

The limitation was articles that were done in the English language and data were extracted from five countries of East Africa only to conduct this systematic review and Meta-analysis and we did not conduct on pooled effect estimate on variables that affect depression in TB patients. As well, all the studies included in this systematic review and Meta-analysis were observational studies in nature as a result; the outcome variable might be affected by confounding variables.

Irrespective of time restriction, all studies conducted in limited East African countries were found, included, and cross-sectional and we did not see pooled effect estimate of different factors.

## Figures and Tables

**Table 2:** The quality and agreed level of bias and level of agreement on the methodological qualities of included studies in a meta-analysis based on sampling, outcome, response rate and method of analysis

Author	Percentage of agreement	Kappa value	Level of agreement	Nos's quality (score on 0 to 9 scale)
Dasa TT	100	1	Almost perfect	9
Ambaw F	100	1	Almost perfect	8
Alemayehu M	100	1	Almost perfect	9
Duko B	100	1	Almost perfect	9
Singh L	100	1	Almost perfect	8
Amuthonl	100	1	Almost perfect	9
Esther L	100	1	Almost perfect	8
Buberwa G	100	1	Almost perfect	8
Alinaitwe R	100	1	Almost perfect	9
